# Diffuse lichen planus-like keratoses and clinical pseudo-progression associated with avelumab treatment for Merkel cell carcinoma, a case report

**DOI:** 10.1186/s12885-019-5759-1

**Published:** 2019-06-04

**Authors:** Michael A. Cardis, Hong Jiang, Julius Strauss, James L. Gulley, Isaac Brownell

**Affiliations:** 1 Medstar Washington Hospital Center/Georgetown University Hospital, Washington, DC USA; 2 Laboratory of Pathology, Center for Cancer Research, National Cancer Institute, National Institutes of Health, Bethesda, MD USA; 3 Laboratory of Tumor Immunology and Biology, National Cancer Institute, National Institutes of Health, Bethesda, MD USA; 4 Genitourinary Malignancies Branch, Laboratory of Tumor Immunology and Biology, Center for Cancer Research, National Cancer Institute, National Institutes of Health, Bethesda, MD USA; 5 Dermatology Branch, National Cancer Institute, National Institutes of Health, 10 Center Drive, 12N240C, Bethesda, MD 20892-1908 USA

**Keywords:** Merkel cell, Immunology, Lichen planus-like keratosis, Immune checkpoint, Drug reactions

## Abstract

**Background:**

Avelumab is an anti-programmed cell death ligand 1 (PD-L1) antibody approved for treatment of Merkel cell carcinoma (MCC) and locally advanced or metastatic urothelial carcinoma. It shares a similar side effect profile to other immune checkpoint inhibitors, including immune-related adverse reactions in the skin. These adverse skin reactions can present as a morbilliform exanthem, lichenoid dermatitis, vitiligo, autoimmune bullous disorder, among others.

**Case presentation:**

We describe a patient with advanced MCC successfully treated with avelumab who developed acute onset diffuse lichen planus-like keratoses (LPLK) at sites of existing seborrheic keratoses (SK) and lentigines. Histopathology of an affected SK revealed papillomatous epidermal hyperplasia with lichenoid interface changes, numerous dyskeratotic keratinocytes and intermittent hypergranulosis. The findings resembled lichen planus (LP) arising in an SK. Onset of the skin symptoms corresponded with an inflammatory cancer response (clinical pseudo-progression), and the eruption improved as overall tumor burden decreased. The patient’s pruritus was treated with topical steroids and cyrotherapy for individual symptomatic lesions.

**Conclusion:**

Diffuse LPLK is a distinct immune-related reaction pattern associated with PD-L1/PD-1 checkpoint blockade. This is an important side effect to be aware of as LPLK frequently mimic keratinocytic neoplasms. Further observation is needed to assess the prevalence and significance of this immune therapy-associated adverse reaction.

## Background

Immune checkpoint inhibitors have emerged as a promising treatment for numerous malignancies, including Merkel cell carcinoma (MCC). With the increased use of immunotherapies, their associated immune-related adverse reactions are becoming increasingly well characterized. Cutaneous reactions are among the most commonly reported side effects of these medications. Herein, we describe a patient who developed extensive lichenoid keratoses as an immune-related adverse reaction during treatment with avelumab for metastatic MCC. We discuss its histopathology, clinical course and potential implications.

## Case presentation

**Fig. 1:**
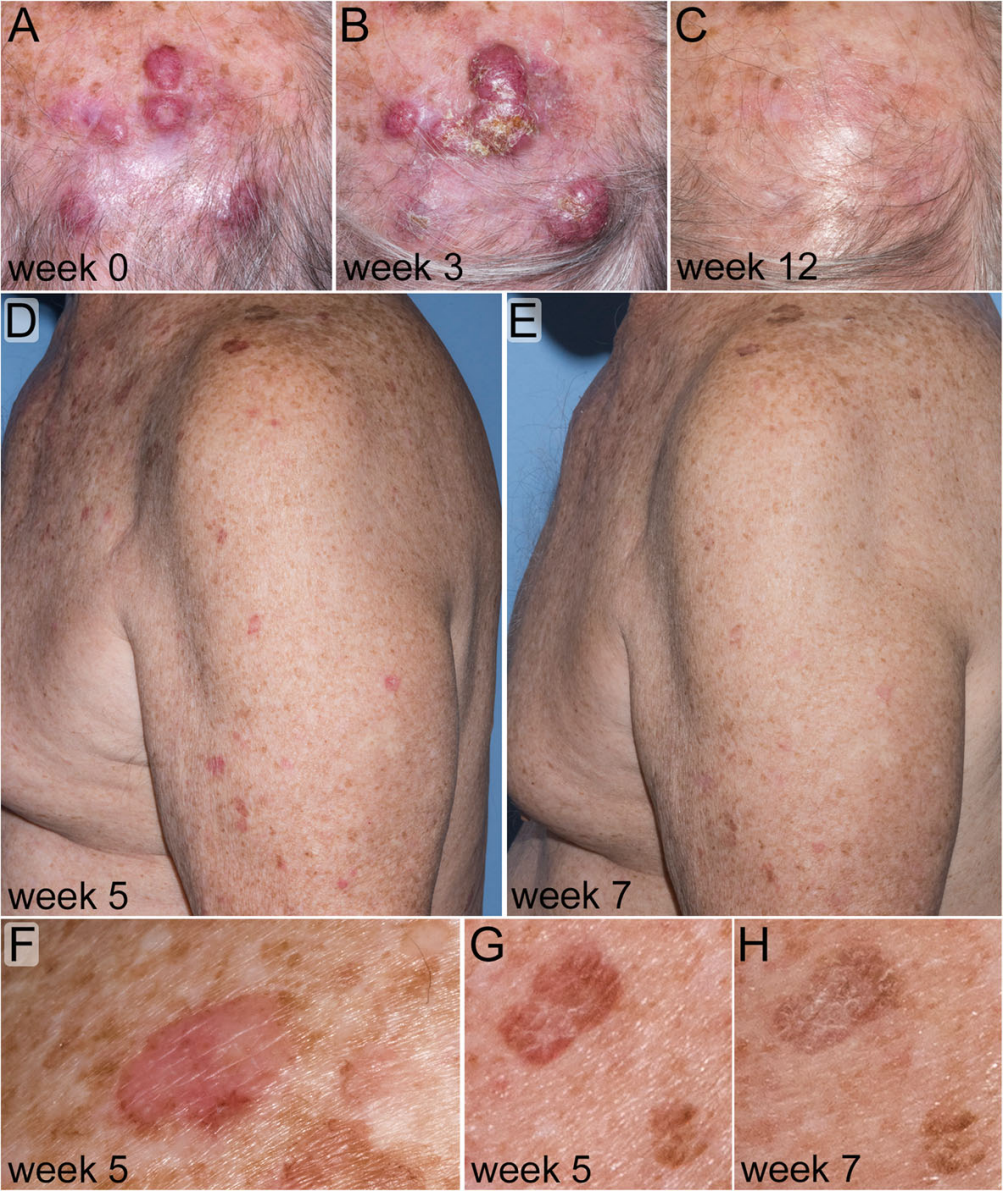
Clinical appearance of tumor and lichen planus-like keratoses (LPLK) in a patient with Merkel cell carcinoma (MCC). **a**: Baseline image of MCC involving the scalp. **b**: Two weeks after the first avelumab infusion MCC lesions were inflamed and slightly enlarged, consistent with pseudo-progression of malignancy. **c**: Complete clinical regression of MCC. **d**, **f** & **g**: Four weeks after starting avelumab the patient had diffuse inflammation of seborrheic keratoses and solar lentigines consistent with LPLK. **e** & **h**: After treatment with topical steroids the LPLK lesions improved

A 73-year-old man with unresectable stage IIIB MCC was referred to the National Institutes of Health for treatment with the monoclonal anti-programmed cell death ligand 1 (PD-L1) antibody avelumab. On physical examination, there were multiple pink to deep red smooth tumors with prominent vasculature on the central scalp (Fig. 1a) and left cervical lymphadenopathy was palpable. Biopsy of a scalp tumor revealed neuroendocrine carcinoma with positive staining for cytokeratin 20 (CK20) and synaptophysin, confirming the diagnosis of MCC. Positron emission tomography/computerized tomography (PET/CT) scanning showed metabolically active cutaneous and subcutaneous nodules on the vertex of the scalp, and multiple metabolically active enlarged cervical and supraclavicular lymph nodes.

The patient was started on avelumab at a dose of 10 mg/kg infused every two weeks. He was pre-medicated with acetaminophen, diphenhydramine and ranitidine. Two weeks after his first infusion his scalp lesions were inflamed and enlarged, consistent with pseudo-progression (Fig. 1b). The scalp tumors and lesions on CT scans subsequently regressed (Fig. 1c).

**Fig. 2:**
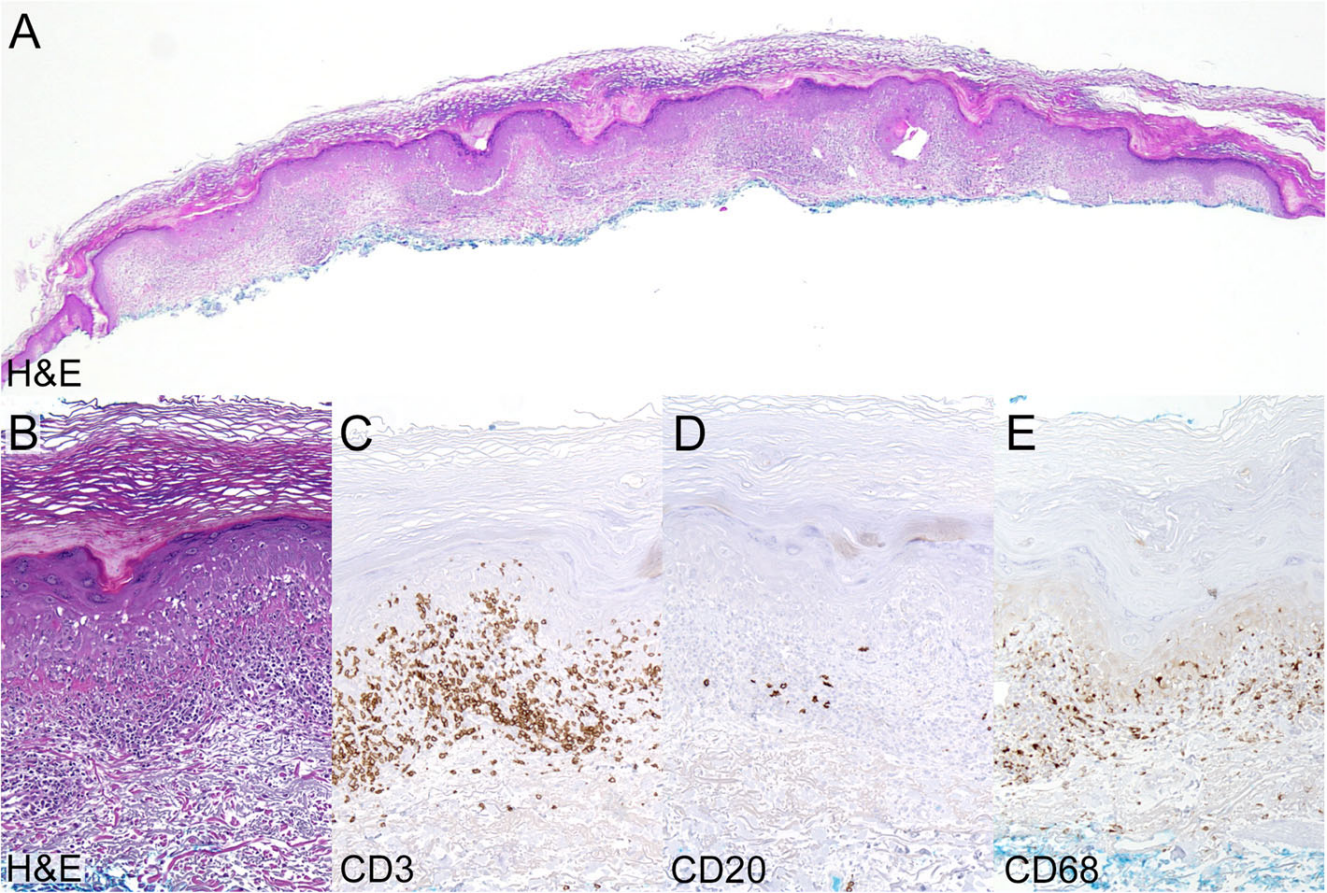
Histology of inflamed skin lesion consistent with LPLK. **a**: Shave biopsy from affected lesion on the right posterior shoulder (Hematoxylin and eosin, original magnification 20x). **b**: High power view. (Hematoxylin and eosin, original magnification 100x). **c**: The lichenoid infiltrate predominately contained T lymphocytes with exocytosis into the epidermis. (CD3 immunoperoxidase stain, original magnification 100x). **d**: The infiltrate contained a paucity of B lymphocytes. (CD20 immunoperoxidase stain, original magnification 100x). **e**: Scattered macrophages were present within the infiltrate. (CD68 immunoperoxidase stain, original magnification 100x)

Between his second and third infusions, the patient developed a pruritic erythematous eruption on the chest, upper back, upper arms and right lower extremity. Examination revealed numerous thin, pink-brown scaly plaques ranging in size from 1.0 cm to 1.5 cm and involving sites of pre-existing seborrheic keratoses (SK) and solar lentigines (Fig. 1d, f & g). A shave biopsy of an affected lesion on the right posterior shoulder was performed and histology demonstrated papillomatous epidermal hyperplasia with hyperkeratosis and focal parakeratosis. The epidermis contained scattered exocytosed lymphocytes associated with mild spongiosis, intermittent hypergranulosis, and copious dyskeratotic keratinocytes. The dermal-epidermal junction was obscured by a lichenoid infiltrate primarily composed of T-lymphocytes. These clinical and histological finding are consistent with lichen planus-like keratosis (Fig. 2a-e). Treatment with topical triamcinolone 0.1% ointment twice daily provided symptomatic relief. Inflammation of affected lesions diminished over the following two weeks (Fig. 1e & h), however, the patient experienced intermittent inflammation in scattered keratoses and lentigines during continued therapy with avelumab. Treatment with cryotherapy was effective at ablating individual symptomatic lesions and resolving the local inflammation.

## Discussion and conclusions

MCC is an aggressive neuroendocrine skin cancer that is often caused by Merkel cell polyomavirus. For patients with metastatic MCC, standard therapies including surgery, chemotherapy, and radiation have not been shown to reduce overall mortality [1]. Fortunately, immune checkpoint inhibitors have proven to be highly effective in treating MCC. Recently, the fully human IgG anti-PD-L1 monoclonal antibody avelumab became the first United States Food and Drug Administration (FDA) approved treatment for MCC [1–3].

Tumor expressed PD-L1 can bind the PD-1 receptor on tumor-infiltrating lymphocytes to induce T-cell tolerance and promote cancer cell survival. In addition to inhibiting the PD-L1/PD-1 immune checkpoint, avelumab can bind tumor cells and induce antibody-dependent cell-mediated cytotoxicity (ADCC). Despite its two potential mechanisms to activate immune responses, avelumab possesses a comparable side effect profile to other PD-L1/PD-1 checkpoint inhibitors [4].

In normal tissues, the PD-L1/PD-1 immune checkpoint helps maintain immunologic homeostasis by inhibiting aberrant autoimmune responses. Blocking PD-L1/PD-1 binding can reverse this inhibition and produce undesirable immune-related adverse events (irAE). Immune-related adverse reactions may affect any organ system, but the skin, gastrointestinal tract, endocrine glands, and liver predominate [5]. The integument is the most commonly affected organ system overall; resulting in a myriad of cutaneous toxicities including pruritus, morbiliform exanthem, vitiligo, psoriasis, lichenoid dermatitis, acneiform eruptions, dermatomyositis, alopecia areata, severe cutaneous adverse reaction (e.g. toxic epidermal necrolysis, drug induced hypersensitivity syndrome), and autoimmune bullous disease. Cutaneous toxicity reportedly occurs in greater than a third of patients, with pruritus and morbiliform exanthem accounting for the majority of eruptions (occurring in up to 20% of total patients treated with immune checkpoint inhibitor therapy) [6, 7].

Lichenoid dermatitis elicited by immune checkpoint inhibitors is a well-established adverse reaction. The precise mechanism is unclear, but is thought to result from disinhibiting an existing immune response, or by either unmasking or creating a neoantigen [8]. It presents as a papulosqaumous eruption composed of coalescing pink to violaceous scaly papules and plaques on the extremities and trunk, resembling generalized lichen planus.

Our patient developed lichenoid inflammation restricted to sites of existing SKs and solar lentigines, producing the abrupt onset of extensive LPLK. The histologic features of our case overlap with that seen in lichenoid drug eruptions, though solely confined to pre-existing keratoses [8]. Histologic features favoring LPLK over traditional lichen planus are the presence of spongiosis, parakeratosis and/or an epidermal architecture resembling a lentigo or SK. Classic LPLK usually present as well-demarcated violaceous to brown, scaly papules or small plaques located on the trunk or extremities, often in middle-aged, fair-skinned women. They almost always occur as a solitary lesion, but have infrequently been reported in a diffuse pattern, particularly in the setting of extensive solar damage [9, 10]. When LPLK manifest as numerous lesions, careful consideration should be given regarding a diagnosis of lichen planus and physical examination findings combined with natural history can help distinguish the two. While LPLK present acutely as erythematous scaly patches and plaques, lichen planus is more insidious and presents with shiny flat-topped polygonal violaceous papules and plaques and typically portends a chronic and recalcitrant disease course. LPLK notoriously mimic keratinocyte cancers such as basal cell carcinoma or Bowen’s disease, but when presenting diffusely may also be confused with classic lichen planus or lichenoid drug eruption [11]. The pathogenesis of LPLK is unknown, but it has been postulated that they represent an inflammatory response targeting a pre-existing epidermal lesion such as SK, lentigo, or actinic keratosis [12]. Extensive inflamed SKs presenting as an immune-mediated adverse event has also been reported in a patient receiving nivolumab for metastatic melanoma [13].

It is unclear if this adverse effect portends any prognostic significance, but it is interesting that the timing of our patient’s eruption coincided with clearance of his MCC, reducing in intensity as the total tumor volume diminished. This may be coincidental, or could indicate that the inflamed keratoses served as a proxy indicator of active antitumoral inflammation. It is noteworthy that Merkel cell polyomavirus gene expression has been described in SKs [14], as have proliferations of Merkel cells [15]. Thus, it is possible that MCC immune epitopes may also be present in SKs, allowing for a cross-reactive immune response.

## Conclusion

Acute onset diffuse LPLK is a newly described immune mediated adverse reaction occurring during treatment of MCC with avelumab. Although the clinical and histologic features resemble that of the irAE lichenoid dermatitis, extensive LPLK has a unique presentation in which lichenoid inflammation is confined to sites of pre-existing SKs and lentigines. It is an important side effect to be aware of since lichenoid keratoses can mimic non-melanoma skin cancers. In our case, the eruption coincided with tumor clearance, was transient, and responded to topical steroids and cryotherapy. When treating with immune checkpoint inhibitors clinicians should take note of pre-existing SKs and lentigines in order to further assess the relationship between their inflammation and treatment response.

## Data Availability

The datasets used and/or analyzed during the current study are available from the corresponding author on reasonable request.

## Abbreviations

irAE: Immune-related adverse event
LP: Lichen planus
LPLK: Lichen planus-like keratosis
MCC: Merkel cell carcinoma
PD-1: Programmed cell death protein 1
PD-L1: Programmed cell death ligand 1
SK: Seborrheic keratosis
